# Decellularized scaffold of cryopreserved rat kidney retains its recellularization potential

**DOI:** 10.1371/journal.pone.0173040

**Published:** 2017-03-07

**Authors:** Baldeep Chani, Veena Puri, Ranbir C. Sobti, Vivekanand Jha, Sanjeev Puri

**Affiliations:** 1 Centre for Stem Cell Tissue Engineering and Biomedical Excellence, Panjab Universtiy, Chandigarh, India; 2 Centre for Systems Biology & Bioinformatics, Panjab Universtiy, Chandigarh, India; 3 Department of Biotechnology, Panjab University, Chandigarh, India; 4 Department of Nephrology, George Institute for Global Health India and University of Oxford, New Delhi, India; 5 Biotechnology Branch, University Institute of Engineering & Technology, Panjab University, Chandigarh, India; University of Kansas Medical Center, UNITED STATES

## Abstract

The multi-cellular nature of renal tissue makes it the most challenging organ for regeneration. Therefore, till date whole organ transplantations remain the definitive treatment for the end stage renal disease (ESRD). The shortage of available organs for the transplantation has, thus, remained a major concern as well as an unsolved problem. In this regard generation of whole organ scaffold through decellularization followed by regeneration of the whole organ by recellularization is being viewed as a potential alternative for generating functional tissues. Despite its growing interest, the optimal processing to achieve functional organ still remains unsolved. The biggest challenge remains is the time line for obtaining kidney. Keeping these facts in mind, we have assessed the effects of cryostorage (3 months) on renal tissue architecture and its potential for decellularization and recellularization in comparison to the freshly isolated kidneys. The light microscopy exploiting different microscopic stains as well as immuno-histochemistry and Scanning electron microscopy (SEM) demonstrated that ECM framework is well retained following kidney cryopreservation. The strength of these structures was reinforced by calculating mechanical stress which confirmed the similarity between the freshly isolated and cryopreserved tissue. The recellularization of these bio-scaffolds, with mesenchymal stem cells quickly repopulated the decellularized structures irrespective of the kidneys status, i.e. freshly isolated or the cryopreserved. The growth pattern employing mesenchymal stem cells demonstrated their equivalent recellularization potential. Based on these observations, it may be concluded that cryopreserved kidneys can be exploited as scaffolds for future development of functional organ.

## Introduction

Once an organ such as the kidney reaches a state of irreversible degradation, organ function must be replaced to ensure survival of the patients. However, the critical shortage of donor organs lead to increased morbidity and mortality for tens of thousands of patients each year. In spite of the fact that some patients are fortunate enough to receive an organ but they are laden with the high risk of chronic rejection and associated morbidity due to simultaneous use of immunosuppressors. A successful regenerative strategy for whole-organ replacement would represent a quantum leap towards the treatment of patients with end-stage organ disease. Although, significant advances have been made in the development of engineered tissues such as blood vessels, urinary bladder and trachea [1–3], as none of these tissues require an intact vascular network that must be connected to the host circulation at the time of implantation. Whole-organ constructs such as kidney, heart, lung, and liver, however require immediate vascular supply and thus represent a challenge for regeneration. However, some of the organs like heart and urinary bladder have been well exploited for regeneration following decellularization [4, 5]. The acellular three-dimensional biologic scaffolds of these organs have subsequently been seeded with either functional parenchymal cells or selected progenitor stem cell populations. Self-assembly of these seeded cells with the aid of a bio-competent three-dimensional matrix, thus, results in the formation of functional tissue in short-term preclinical animal models [6, 7]. This approach provides the opportunity for direct connection to the patient vasculature in either an orthotopic or heterotopic locations. Fortunately, advances in the field of regenerative medicine have now been seen as a renewed hope of regenerating the renal tissue.

Despite growing interest on the potential use of decellularized whole kidney as 3-dimensional scaffolds for *ex vivo* kidney tissue generation, number of questions remain unanswered. As demonstrated in different tissue types such as skin, muscle, bladder, and others the successful use of biologic scaffolds has already entered clinical practice. Methods of optimal storage have been suggested for other organs like heart valves, trachea etc but not yet clearly delineated for kidney. Further, it is unclear whether kidneys need to be obtained immediately or may be usable even if harvested several days post-mortem, a situation mimicking potential procurement of human kidney from autopsy. The time frame of tissue recovery from autopsy needs to be addressed. It will even be more challenging to envisage suitability of the donor kidney for decellularization followed by recellularization with regenerated function. These certainly represent some of the key challenges which will come up once the basic understanding has been achieved in the experimental animals.

Keeping this in mind, we have, therefore, assessed the effects of prolonged cryostorage (3 months), on architecture and extracellular matrix (ECM) protein characteristics of the decellularized rat kidneys. The observations pointed to similarity in the extent of decellularization when freshly isolated kidneys were compared with cryostored structures. This observation was specifically relied upon the similarity in the histological appearance, retention of ECM and intracellular proteins as assessed by immuno-histochemistry in spite of reduced mechanical strength of the cryostored kidney. The differences, although subtle, suggested that cryopreservation can be exploited for generation of whole kidney bio-scaffold for renal tissue regeneration.

## Material and methods

### Ethics statement

The experimental protocols used in our study were approved by the Institutional Animal Ethics Committee (IAEC) (Approval ID:PU/IAEC/S/14/167) of Panjab University, Chandigarh, India and performed in accordance with the guidelines of Committee for the Purpose of Control and Supervision of Experiments on Animals (CPCSEA), Government of India. All efforts were made to minimize the suffering of animals.

### Organ preparation

Twenty four (24) kidneys were harvested from 12 female 250–350-g Sprague-Dawley rats and divided into three groups (Group I, II, III) of 8 kidneys each. Group I consisted of freshly isolated kidneys from rat (SD strain) served as control as no decellularization treatment was given to this group. Group II consisted of freshly isolated kidneys which underwent decellularization treatment immediately after isolation (no cryopreservation). Group III consisted of kidneys which were cryopreserved for 3 months after which these underwent decellularization treatment. Before any treatment was given the organs were thoroughly perfused using normal saline along with anticoagulant treatment using heparin. For this the cannula was inserted in the renal artery as an inlet for perfusion and ureter as an outlet.

### Cryopreservation process and reconditioning

For cryopreservation, the kidneys were immersed in 100 ml of culture medium DMEM containing 10% dimethyl sulfoxide (DMSO) and 10% fetal bovine serum (FBS). The organs were then packed in aluminum foils, sealed and kept at temperature of 2–8°C for 30 minutes, followed by their transfer to -80°C deep freezer for 24 hr and finally stored for 3 months at a temperature of liquid nitrogen vapor (-180°C).

#### Reconditioning

Before start of the decellularization process all the kidneys were equilibrated in a similar way as was done for the cryopreserved kidneys. Briefly the cryopreserved kidneys were thawed in a sterile container maintained in a water bath at 37°C, and cryoprotectant solution was replaced with DMEM containing 10% FBS then rinsed thoroughly with deionized (DI) water.

### Decellularization process

Decellularization was an adaptation of procedure described by Sullivan [8]. Briefly, the kidneys were perfused through renal artery with continuous fluid delivery using a Gilson peristaltic pump with phosphate buffered saline (PBS) followed by a decellularization solution consisting of 1% SDS (Sodium dodecyl sulfate) diluted in distilled (flow rate 18ml /hr) water at room temperature for a period of 48 hours. The kidneys were then washed with a solution of deoxyribonuclease I (0.2 mg/ml) and 10 mM MgCl_2_ in PBS at room temperature for a period of 16 hr to ensure complete removal of detergents and nuclear material. Finally the structures were rinsed with PBS to remove deoxyribonuclease I and MgCl_2_ (flow rate 18 ml/hr).

### Light microscopy and scanning electron microscopy of scaffolds

#### Light microscopy

Kidneys from Group I, II and III were fixed in 10% buffered formalin and embedded in paraffin, 5μm thick sections were then cut for preparing slides and used for differential staining. Hematoxylin and Eosin staining [nuclei (blue)/remaining tissue (pink)] was performed to assess the degree of cell removal and preservation of the ECM architecture. To evaluate the presence of collagen, Masson’s Trichrome staining was performed [collagen (blue)/nuclei (black)/cytoplasm (red)] respectively. To determine proteoglycans content, Alcian Blue stain was used (blue for proteoglycans). To assess glycoproteins content, the Periodic acid-Schiff (PAS) staining was performed (pink for glycoproteins).

#### Immunohistochemistry

Immunohistochemistry was performed to evaluate the retention of the key basement membrane proteins viz. collagen IV and laminin following decellularization procedure. Kidney tissues were fixed in 4% paraformaldehyde prepared in phosphate buffered saline (PBS), and embedded into paraffin wax at 58–60°C. Sections corresponding to 5μm thickness were prepared and rehydrated with serial alcohol dilutions (100%, 90%, 70%, 50% and 30%) for 2 minutes each. These sections were then blocked with 2% BSA in PBS for 30 minutes and antigen retrieval was carried out in 10 mM citrate buffer (pH 6.0) using microwave. Following antigen retrieval the sections were incubated overnight with primary antibodies Collagen1V (Abcam, 1:200), laminin (Boster Bio, 1:200) at 4°C in a humidified chamber. For collagen, the slides were washed with PBS and incubated with goat anti-rabbit IgG FITC conjugated secondary antibody (Merck Biosciences) for 2 hrs. The sections were counter-stained with propidium iodide (BD Biosciences) to visualize the nuclei (red) using Nikon Eclipse 80i microscope. For laminin, the slides were incubated in 0.3% H_2_O_2_ in Tris buffered saline (TBS) for 15 min to block endogenous peroxidase and then incubated with a goat anti-rabbit IgG HRP conjugate secondary antibody (Santa Cruz, 1:20,000) for 2 hrs. These slides were then exposed to DAB-Substrate (200 μl) to completely cover the section and incubated for 5 min at room temperature. Each step of staining was followed by rinsing with tap water and counterstained with Hematoxylin (blue). The stained slides were rinsed under running tap water for a period of 5 min. The slides were dehydrated by incubating in ethanol for 3 min each in the serial order as 70%, 80%, 96%, 100% and finally in 1:1 absolute ethanol and Xylol. The slides were then examined using Nikon Eclipse 80i microscope.

#### Scanning electron microscopy

Different tissue samples following each procedure from group 1, II and III were first fixed using glutaraldehyde for 24 hours at 4°C and were then dehydrated in graded ethanol (30%, 50%, 70%, 90% and 100%). The samples were then dried using a CO_2_ critical point dryer. The samples were loaded onto aluminum stubs, coated with gold and examined under a scanning electron microscope (JSM 6100, JEOL, USA).

### Biomechanical test

The specimens were subjected to uniaxial tension until failure. This test records the stress (tensile strength) “s” versus strain “ε”; the highest point of the stress & strain curve is the ultimate tensile strength (UTS). The ratio of stress to strain (Young’s modulus, E), which is a measure of the stiffness of an elastic material, was also calculated. Mechanical tests were performed with the application of uniaxial tension in an Instron 5565 at room temperature (20°C). Specimens were loaded at a constant tension rate of 20 mm/min. Stress—strain relationships, ultimate tensile strength (UTS), defined as maximum stress that a material could withstand until it breaks, and tensile modulus were obtained for all the groups i.e. I, II, III and graphs plotted. Experiment was performed in triplicates for each evaluated tissue.

### Cell maintenance

The murine C_3_H_10_T½ cells were procured from NCCS Pune, India [9]. Cell line was maintained in growth medium DMEM (with high glucose content), supplemented with 10% FBS (heat inactivated), 100 U penicillin, and 100 μg/ml streptomycin. Cells were cultured in 75 cm^2^ polystyrene flasks (BD, India) in a controlled atmosphere at 37°C and 5% CO_2_. Cells were sub-passaged every 3–4 days upon reaching confluence 90%.

### Renal scaffold sterilization

Following decellularization, whole kidney scaffolds were placed in PBS solution supplemented with penicillin (100U/ml), streptomycin (1mg/ml), deoxyribonuclease I (0.2mg/ml) for 24 hours at 4°C. The solution (1X PBS containing 1% antibiotics) was replaced after every 5 hr for 24 hours to flush out residual deoxyribonuclease I. The sterilized decellularized matrix was stored in PBS at 4°C prior to further processing and cell seeding. Additionally, the entire scaffold was further exposed to UV light under the laminar flow hood. At first the scaffold was dipped in PBS plus antibiotic and kept in the UV light for 1–2 hours and then the scaffolds were placed in DMEM and sterilized by UV light for 1 hour.

### Cytotoxicity studies

#### a) Biocompatibility study

To determine whether the decellularized kidney ECM contain any leachable toxins or not, a cell-scaffold biocompatibilty experiment was carried out. For this the decellularized kidneys (fresh and cryopreserved) were washed overnight with PBS containing solution of 1% penicillin /streptomycin. Thin sections (5.0 mm) of decellularized kidneys, from both the freshly isolated and cryopreserved kidneys were placed along with 1x 10^5^ undifferentiated murine C_3_H_10_T½ cells per well of six well plate to analyze the extent of any soluble and contact toxicity.

#### b) MTT assay

The cytotoxic activity of kidney decellularized ECM for C_3_H_10_T½ cells was determined by MTT assay. In this assay, the cells were cultured in full growth medium along with both group II and group III decellularized scaffolds for a period of 24 hrs and compared against the cells cultured in full growth medium without any scaffold (as Positive control) and cells cultured in full growth medium with 1% SDS (decellularization medium) taken as negative control.

### Seeding and recellularization culture

#### Cell preparation

Upon reaching 80–90 confluence state murine C_3_H_10_T½ cells were trypsinized and maintained in full growth medium DMEM (with high glucose content), supplemented with 10% FBS (heat inactivated), 100 U penicillin, and 100 μg/ml streptomycin.

### Seeding and recellularization analysis

About 2x10^6^ continuously growing C_3_H_10_T½ cell were concentrated and resuspended in 2 ml of medium and injected either via the cannula (flow rate 6.0 ml/hr) or directly through the walls of the both freshly decellularized kidney and decellularized structures of cryopreserved kidney scaffolds at regular spacing (~5mm) with an 18 gauge needle. The tissue was placed in static culture at 37°C with full growth medium. The tissue was cultured for 6 days. After culture, tissues were brought into a biological safety cabinet at different time points at (2^nd^, 4^th^, 6^th^ day) and the tissue was removed for dissection. A randomly selected portion of the both recellularized scaffolds were excised. The tissue sections were rinsed 3 times with media and PBS followed by final washing in PBS. The sample was fixed in 4% paraformaldehyde and embedded in paraffin, and 5 μm-thick sections were obtained and used further to confirm through H&E staining, whether these cells repopulated the scaffolds.

### Statistical analysis

Statistical analysis was performed using one way ANOVA from Graph Pad Prism 3.0 (Graph Pad Software, Inc., San Diego, CA). Data presented here were expressed as the mean ±SEM. P values <0.05 were considered to be statistically significant.

## Results

### Cryopreserved kidneys retain decellularization potential similar to that of freshly isolated kidneys

**Fig 1:**
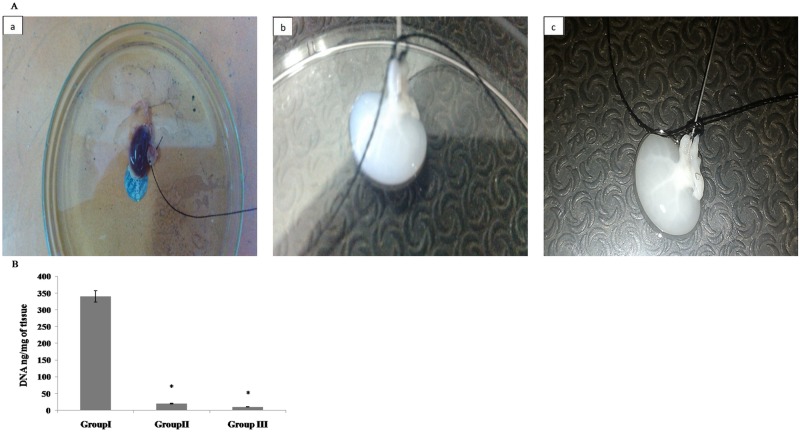
A. Perfusion with 1% SDS completely decellularized the kidney structures. The collateral kidneys from rats were isolated and perfused with normal saline prior to perfusion with 1% SDS diluted in distilled water at room temperature for a period of 48 hours. The kidneys were then washed with a solution of deoxyribonuclease I (0.2 mg/ml) and 10 mM MgCl_2_ in PBS at room temperature for a period of 16 h to ensure complete removal of detergents and nuclear material. Finally the structures were rinsed with PBS to remove deoxyribonuclease 1 and MgCl_2_ The photomicrographs in **Panel-A** represents **a)** control freshly isolated kidney representing group I, **b)** decellularized freshly isolated kidney representing group II, and **c)** decellularized kidney from 3 months cryopreserved kidneys representing group III. White transparent appearance of both kidneys from groups I & II shows that kidneys have undergone complete loss of parenchyma in comparison to freshly isolated kidneys group I. **Panel B** represents a graph of total DNA content analyzed in control kidneys, group I, following decellularization of freshly isolated kidneys, group II and cryostroed kidneys, group III. Based on the DNA quantification it was observed that 1% SDS was sufficient to decellularize the kidneys to similar extent whether the kidneys were freshly isolated or cryostored. Data are expressed as mean ±SEM. Based on ANOVA, significant differences among groups II, III w.r.t. control group I are indicated at *p<0.05.

The effect of cryopreservation on renal tissue decellularization, was observed using kidneys from group I, II & III. All the kidneys were kept at 37°C for period of 15 minutes after which organs were decellularized using a cocktail of detergent as mentioned in materials and methods. Following 12 hours of perfusion with detergent (1% SDS), the decellularization was partial but by 36 hours a complete decellularization was achieved. As shown in Fig 1A the kidneys turned transparent whether from group II or group III in comparison to control kidneys from group I and cryopreserved kidney before decellularization after thawing (S1b Fig). The freshly isolated kidneys from group II underwent 39% decrease (1.28g to 0.78 g) in the kidney weight after 48 hours of decellularization. In case of cryopreserved kidneys from group III the weight reduction was observed at two levels, first when the kidneys were thawed a reduction corresponding to 27% (from 1.35 g to 0.98 g) and when this thawed tissue was decellularized a further reduction in the weight to ~ 30% (0.98 g to 0.68 g). Overall at the end of decellularization, the cryopreserved kidneys underwent a total reduction in weight to ~ 49% with respect to original weight of kidney (from 1.35 g to 0.68 g). In order to further validate, this reduction in the extent of decellularization was reconfirmed by analyzing DNA content. This was analyzed after extraction of any residual DNA following decellularization in comparison to control kidney. As shown in Fig 1B, almost comparable level of decellularization was achieved as reflected by similar level of reduction in DNA content, i.e. ~96% and ~98% in Group II & III, respectively.

### ECM composition & architecture of decellularized kidney remain refractory to cryopreservation

**Fig 2:**
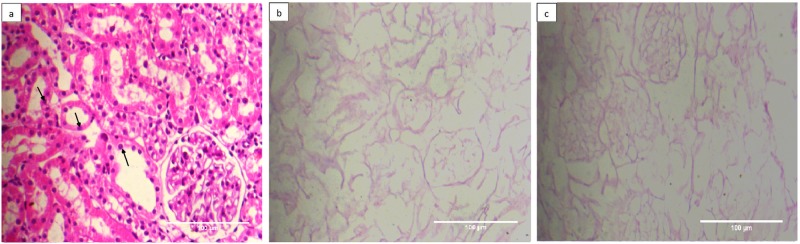
Cryostorage retains the basic kidney micro-architecture and undergoes decellularization to a similar level as in freshly isolated kidneys. Photomicrographs represented H&E staining of **a)** control kidneys from group, I **b)** decellularized structure from freshly isolated kidney, group II, **c)** decellularized structure from cryostored kidney, group III at 200X. Well preserved ECM architecture with no evidence of residual nuclei or intact cells as resolved by Hematoxylin (blue stained nuclei) and Eosin (pink stained intracellular proteins), could be seen in both the cryostored kidneys (group III and freshly isolated kidneys group II). Black arrow represents renal cells.

**Fig 3:**
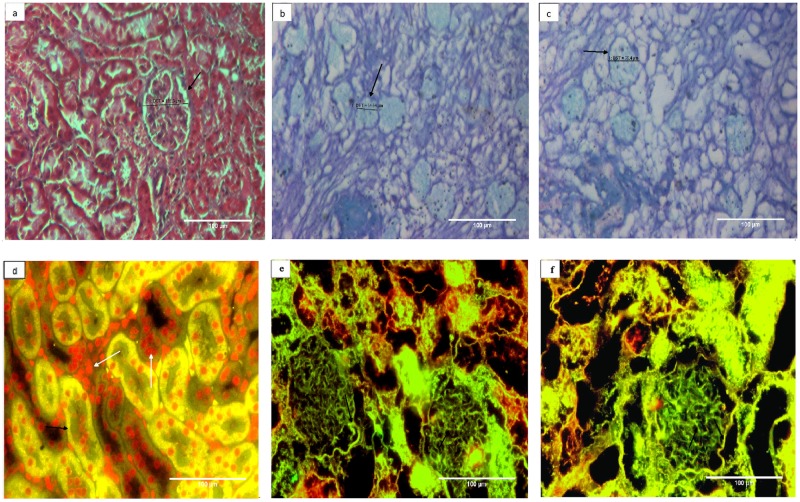
Collagen content of basement membrane is well retained whether or not the kidneys were cryostored prior to decellularization. Both Masson’s trichrome staining **(photomicrogrpahs a-c)** and immunohistochemistry using anti collagen IV antisera **(photomicrograph d-f)** were used to analyze the effect of cryostorage on collagen status of the decellularized structures. Masson’s trichrome stains collagen blue, cellular component red and nuclei black. The blue stained structures (black arrows) showed well retained collagen component of the ECM from **a)** represented control kidney from group I, **b)** decellularized kidney from group II, **c)** decellularized kidney from group III. Almost similar extent of staining patterns among group II and III demonstrated that decellularization process of kidneys did not affect the collagen content whether the tissues were cryopreserved or not. Absence of red and black stain re-confirms the absence of any cellular material and nucleus in these decellularized kidneys. With respect to immunohistochemistry **(fig d-f)** green fluorescence represented collagen IV and the red fluorescence indicated cell nucleus (Propidium iodide, PI stain). Photomicrographs represented **d)** control kidney from group I, **e)** decellularized structures from freshly isolated kidney, group II and **f)** decellularized structure from cryostored kidney, group III at 200X. Where-ever shown, the white arrows represent renal cells (red fluorescence) and black arrows represent collagen 1V (green fluorescence). It is clear from the Figs that after decellularization overall presence of ECM component collagen IV in glomerulus and kidney structure (black arrow) is preserved and cells are completely removed as no red fluorescence observed when compared with control.

Histological analysis using H & E, Masson’s Trichrome, Alcian Blue & PAS staining, demonstrated a similarity in architecture of the decellularized kidneys whether cryopreserved or not. The observations demonstrated well preserved cellular architecture of renal tissue following decellularization. The photomicrographs in Fig 2 demonstrated that in comparison to control kidney from group I (Fig 2a), the detergent induced decellularization of both the freshly isolated kidney from group II (Fig 2b) and the cryopreserved kidney from group III (Fig 2c) was successfully achieved i.e. a complete removal of cellular material. No discernable differences could be seen when the decellularized structures of cryopreserved kidney (group III, Fig 2c) were compared with freshly isolated kidney (group II, Fig 2b). Moreover, the cryopreservation also did not disturb extra-cellular architecture at different skeletal regions of kidney tissue i.e. glomerulus and tubular structures, as these structures were found to be well retained and comparable as shown in Fig 2c to 2b. The ECM components of the decellularized structure following cryopreservation retained web like appearance similar to that of the freshly isolated kidneys (compare Fig 2c with 2b). Moreover, triple staining employing Masson’s Trichrome demonstrated absence of any pink and purple color (nuclear stain) and similar intensity of blue color (collagen, Fig 3b & 3c, black arrows) in decellularized structures, in either fresh or cryopreserved kidneys. These results further reiterated that cryopreservation did not alter the collagen content (Fig 3). However, slight loosening of ECM component in cryopreserved decellularized kidneys (Fig 3c, black arrow) was evident in comparison with freshly isolated decellularized kidneys. The loosening of ECM following cryopreservation in comparison to fresh kidneys was also evidenced by variation on the overall diameter of different renal structures viz. tubules and glomeruli, respectively, following decellularization (S2 and S3 Figs).

**Fig 4:**
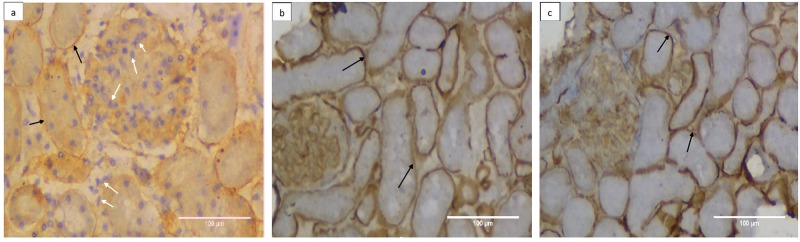
Cryopreserevd kidneys retain their laminin content even after decellularization. Laminin immunohistostaining was carried out using anti laminin sera followed by FITC conjugated secondary IgG representing **a)** control kidney from group I, **b)** decellularized structures from freshly isolated kidney, group II and **c)** decellularized structure from cryostored kidney, group III at 200X. Where ever shown, the white arrows represented renal cells (blue stain) with black arrows representing laminin (brown stain). The cryostorage for 3 month minimally affected the histological appearance and overall presence of ECM component in the decellularized structure, group III in comparison to freshly de-celularized structure, group II.

In order to further validate these observations specific immunohistochemical staining for selected ECM proteins, employing antisera for collagen IV (Fig 3d–3f) or laminin (Fig 4) & counter stained with Propidium Iodide (PI) and Hematoxylin respectively, was carried out. The photomicrographs in Fig 3d–3f represented the meshwork of collagen (green fluorescence, black arrow) uniformly distributed in renal tissue. Absence of any red stain (PI, Fig 3e and 3f) or blue stain (Hematoxylin Fig 4b and 4c) reflecting loss of nuclei, reaffirmed completion of decellularization process. The cryopreservation also did not affect the collagen network as evident by uniform structure assortment seen in decellularized tissue from freshly isolated kidney group I (Fig 3d, 3e and 3f, green fluorescence, black arrows) in comparison with decellularized scaffold of cryopreserved kidney group III. Similar observations were also seen when the laminin protein of scaffold was immunostained with laminin antisera represented by brown stain (Fig 4, black arrows). Cryopreservation of the kidneys for 3 month did not affect the distribution of laminin in tissue upon comparing freshly decellularized tissue group I with decellularized scaffold of cryopreserved kidneys group III (Fig 4b & 4c). Absence of blue color stain (Hematoxylin) in these decellularized structures in comparison to control kidneys, additionally, supported a complete removal of cellular components.

**Fig 5:**
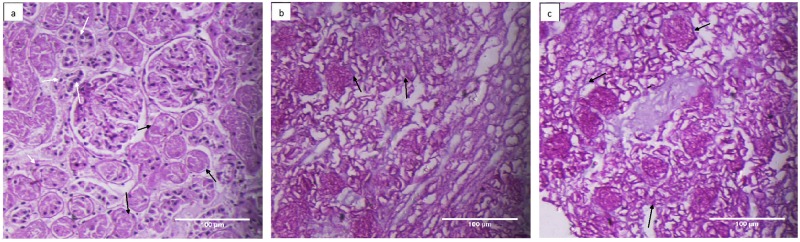
Cellular level of Glycosaminoglaycans (GAGs) underwent a loss following decellularization and not the GAGs present in Extracellular matrix. Alcian blue staining was used to localize the GAGs as shown in photomicrographs **a)** control kidney from group I, **b)** decellularized structures from freshly isolated kidney, group II and **c)** decellularized structure from cryostored kidney, group III at 200X. Where ever shown, the white arrows represented renal cells (black stain) and black arrows represented GAG staining (blue stain). A low content of staining was observed in the decellularized structures from group II and III in comparison to wild type kidneys, group I.

**Fig 6:**
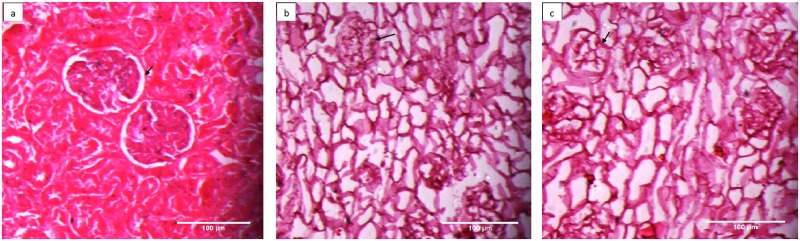
Glycoproteins of tubular basement membrane remained well preserved in decellularized structures from cryostored or freshly isolated kidneys. The glycoproteins were analyzed using PAS staining from **a)** control kidney from group I, **b)** decellularized kidney from fresh kidneys, group II and **c)** decellularized kidney from cryostored kidneys, group III at 200X. Black arrows represent glycoproteins, which remained uniformly stained whether the decellularized structures were obtained from freshly isolated kidneys or cryostored kidneys.

The Glycosaminoglycans (GAG), the important component of renal tissue ECM were stained using Alcian Blue (Fig 5a). The blue staining here is representative of presence of GAG as shown in Fig 5 (black arrows). Since majority of the GAGs are part of the ECM component of the cellular structure a sharp difference between the GAGs staining was observed for cellularized structure (Fig 5a; arrows show renal tissue parenchymal GAGS along with GAGs within ECM, black arrows) and decellularized structures (Fig 5b & 5c, black arrows). However the cryopreservation did not perturb the pattern of GAGs staining of the decellularized structures (compare Fig 5b & 5c). Further, the architecture of both Glomerular Basement Membrane (GBM) and bowman capsules, discerned through PAS staining (dark pink colour Fig 6, black arrows) was found to be well retained, in the decellularized scaffolds, irrespective of the fact that whether the kidneys were cryopreserved or not.

**Fig 7:**
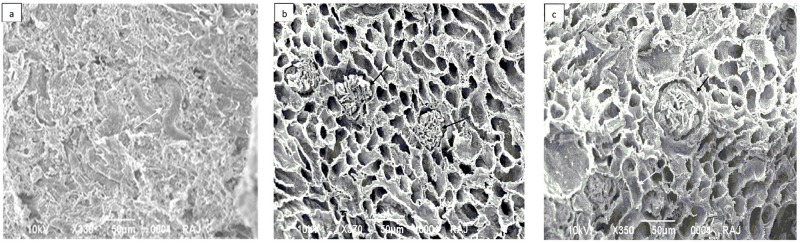
Scanning electron micrograph confirmed complete removal of cells in both cases. Photomicrographs represented **a)** control kidney from group I, **b)** decellularized kidney from freshly isolated kidneys, group II and **c)** decellularized kidney from cryostored kidneys, group III. The acellular glomerulus (black arrow) and adjacent tubules (white arrow) show continuous basement membrane architecture after SDS protocol of decellularization in both the groups i.e. group II & III as compared to control.

The Scanning electron microscopy was used to evaluate the impact of cryopreservation following decellularization on the 3D-architecture and micro-structure of the ECM (Fig 7). The overall kidney tissue micro-structure appeared to be well maintained whether the decellularization was carried out following 3 months of cryopreservation group II (Fig 7c) or in the freshly isolated kidneys, group I (Fig 7b). The architectural structures such as extracellular matrix exoskeleton along with distinct glomerulus (Fig 7, black arrows) could be seen as completely devoid of cellular components, so are the tubular structures (Fig 7 white arrows) which could be seen as in their typical tubular features and distribution within the scaffold (Fig 7b & 7c). The native renal tissue architecture was characterized by a three-dimensional network of connective tissue fibers arranged in a honeycomb-like structure (Fig 7b & 7c). Overall the surface morphology showing tissue microanatomy was found to be well preserved despite 3 months of cryopreservation of the tissue (Fig 7c).

The decellularized kidney structure following cryopreservation retained the mechanical and elastic nature in comparison to the decellularized structures of freshly isolated kidney. The forced compression of these organs immediately re-expanded upon release of force. This mechanical strength was analyzed by calculating the ratio between stress and strain.

### Mechanical strength

**Fig 8:**
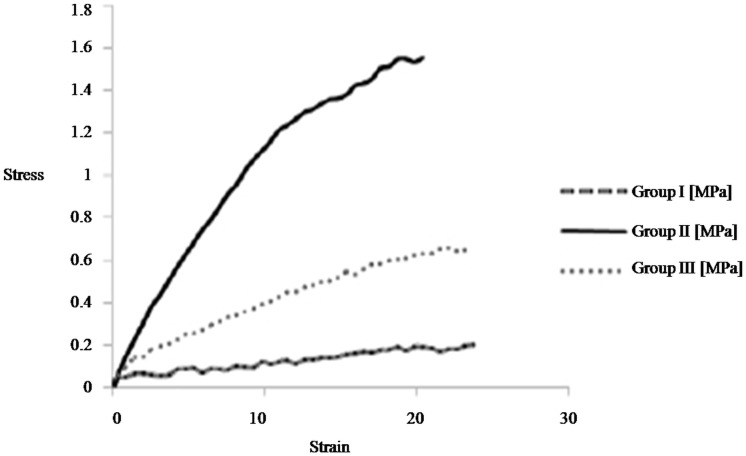
The well preserved ECM maintains the mechanical strength of the decellularized structure from cryostored kidneys. Stress & Strain curve demonstrate progressive rise in tensile strength as Young’s modulus increase after decellularization in both group II, III kidneys when compared with control kidneys from group I. The tensile strength is function of the ECM components of the decellularized structures. Higher tensile strength of the decellularized structures from cryostored kidneys (group III) in comparison to freshly isolated decellularized kidneys (group) is indicative of well preserved ECM following cryopreservation.

Assessment of the mechanical properties of the both Group II, III showed a progressive rise in tensile strength after decellularization (Fig 8). Consequently, the maximum stress that the decellularized kidneys whether cryopreserved or not, could withstand before rupture was higher as compared to the control kidney. The maximum load withstood by cryostored kidney and freshly isolated kidney was ~7.7 fold (1.55 Mpa) and ~ 3 fold (0.66 Mpa), respectively higher as compared to control which withstood only 0.20 Mpa load. These results in Fig 8 thus reflected highest tensile strength of the freshly decellularized structures that reduced almost 2 fold when these structures were first cryopreserved. These changes are justified as freezing/thawing may lead to more exhasustive loss of nuclear and cellular components. Moreover, freeze/thaw affecting the ECM can also not be ruled out. But both decellularized scaffolds showed higher tensile strength than the wild type kidneys as decellularized structures were left with only extracellular matrix making them more flexible so as to withstand more loads before giving way. Overall, the results showed that the cryopreservation though, did affect the tensile strength of the decellularized structures considerably compared to non-cryopreserved kidneys, but it was still far above than the wild type kidneys.

### Cryopreservation retains the growth pattern and viability of cells on decellularized scaffolds

**Fig 9:**
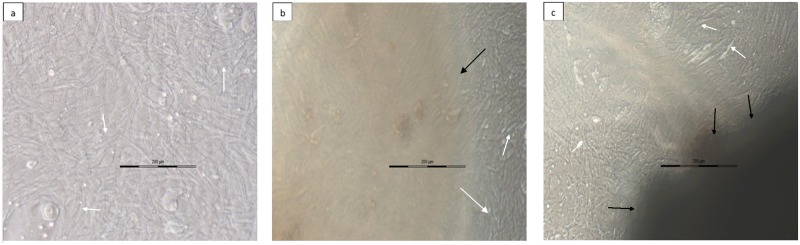
Decellularized renal scaffold from cryostored kidneys retained the cells growth characteristics. Photomicrographs following phase contrast microscopy represented **a)** control kidney from group I, **b)** decellularized kidney from freshly isolated kidneys, group II and **c)** decellularized kidney from cryostored kidneys, group III. As shown the acellular matrix appeared to be biocompatible when murine C_3_H_10_T½ cells were seeded *in vitro*. The cells showed potential for the attachment and underwent proliferation and remained viable when cultured with the decellularized structures whether kidneys were obtained following 3 months of cryostorage or freshly isolated kidneys. Black arrows represent kidney scaffold, white arrows represent attached cells.

The decellularized scaffolds supported the growth of murine C_3_H_10_T½ cells over a period of 4 days equally both in case of freshly isolated kidneys from group II (Fig 9b) and the cryopresereved kidneys tissue from group III (Fig 9c) in comparison to control group I (Fig 9a). After 4 days culturing the cells were found to be growing at similar rate when the decellularized structures of cryopreserved or the freshly isolated kidneys were compared with the cells growing on plastic ware (Fig 9c, 9b & 9a, respectively). During these four days neither any contact toxicity nor any changes in the growth pattern was observed. Cells attained confluence at same period and maintained their fibroblastic morphology whether seeded on plasticware in presence or absence of scaffold. As the measurement of the growth pattern of the cells on scaffolds was difficult under the current circumstances, therefore, it was visualized through growing the cells along with scaffolds in the same well. The observations suggested that cryopreserving the kidneys do not interfere with growth of the cells and the decellularized structure generated from these kidneys also did not cause any toxicity to cells (Fig 9a, 9b & 9c).

#### MTT assay

**Fig 10:**
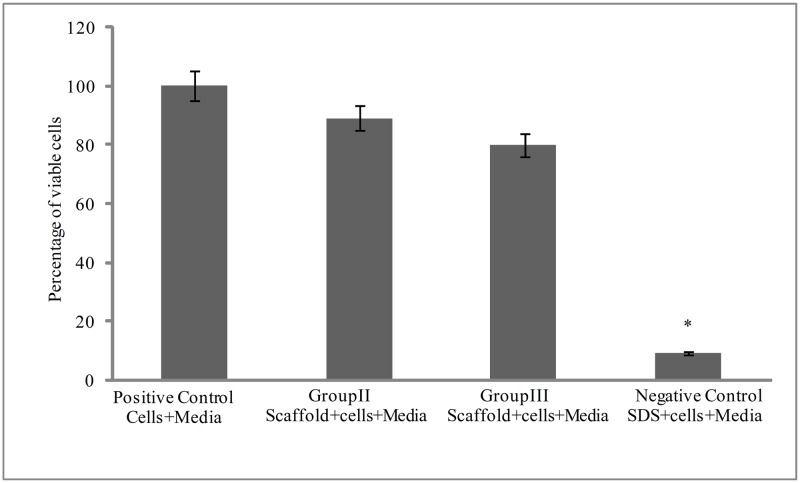
Decellularized scaffold were found to be nontoxic to murine C_3_H_10_T½ cells. The MTT assay was performed to check if the SDS based decellularization of the kidneys bioscaffolds remained nontoxic to mesenchymal stem cells. The assay was performed under different conditions i.e. using only cells and media (i.e. without scaffold) as a positive control, cells with SDS in media as negative control, cells incubated with freshly isolated decellularized kidnyes (group II), and decellularized structures from cryostored kidnyes (group III). As shown in the graph, the SDS inhibited the growth of cells and for negative control the cell viability was only 9.29% while its was found to be 89.2% for the group II) and approximately 80% for the group III). Absorbance was measured in replicates of six and the calculated standard error of the mean (SEM) plotted error bars. Data are expressed as mean ±SEM. Based on ANOVA, significant differences among groups II, III w.r.t. control group I are indicated at *p<0.05.

Non-cytotoxic nature of decellularized scaffold is also reassured through MTT Assay. The MTT assay showed that in positive control when only cells and media were present (i.e. without scaffold), growth of C_3_H_10_T½ cells was normal. This represented as a positive control and the absorbance values of formazone formed in positive control were taken as 100% (Fig 10). Upon comparing it with the negative control, (the decellularization medium containing SDS), it was observed that SDS inhibited the growth of cells, and only 9% cells were found to be viable. While the cell viability was found to be ~89.2% when the C_3_H_10_T½ cells were incubated with decellularized scaffold matrix and ~ 80% viable cells were seen with decellularized structure generated out of cryostored kidneys, following thorough washing (Fig 10). These observations suggested that, both types of de-celluarized scaffolds were almost free from any residual decellularization agent (1%SDS), which can be responsible for any effect on the loss of viability.

#### Cryopreserved scaffold retain the recellularization potential

**Fig 11:**
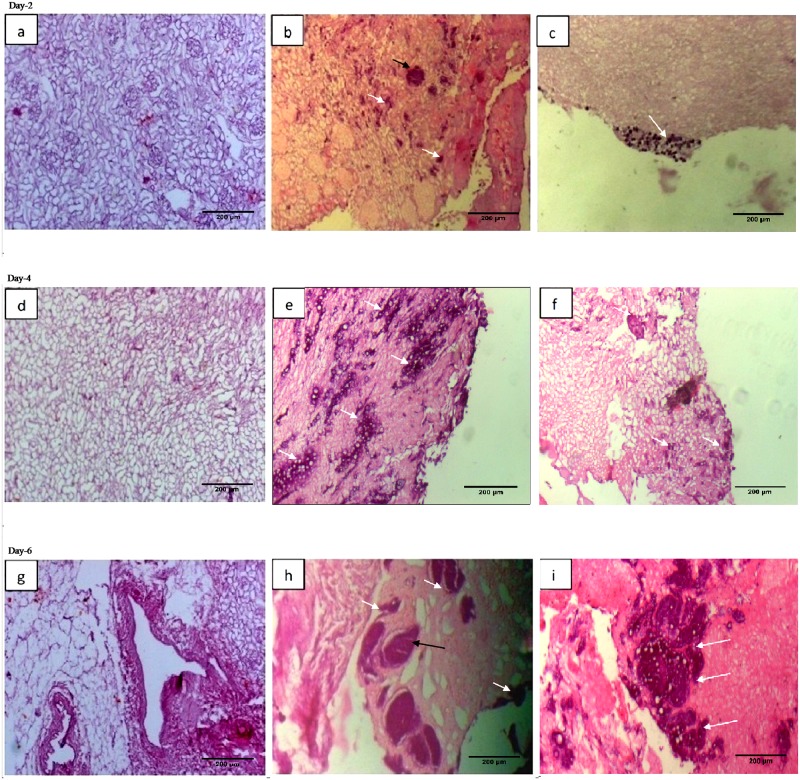
The decellularized structures from cryostored kidneys retained the recellularization potential similar to that of freshly decellularized kidneys. Approximately 2 million C_3_H_10_T½ cells were used for the recellularization. The recellularization of the scaffolds was analyzed at different time intervals Photomicrographs **a), d), g)** represented decellularized kidney scaffolds without recellularization as control, photomicrographs **b), e), h)** represented recellularized kidney scaffolds from group II and photomicrographs **c), f), i)** represented recellularized kidney scaffolds from group III for day 2, 4 and 6, respectively. The cells movement was found to be increased to more deeper areas at different time points following recellularization. Black arrows represented cells repopulating in glomerulus and white arrows represented cells repopulating in different renal tissue areas.

Approximately 2 million cells of C_3_H_10_T½ were taken and re -suspended in 1ml of full growth medium and injected in renal artery via the cannula (23G) or at other times directly on to the scaffolds with a 26 gauge needle. Following 2 days of cellular loading, most of the cells were initially captured in the outer regions as we can see in Fig 11c (black arrows). The pattern of distribution of the injected cells was observed into vascular structures and within the glomerulus (Fig 11b & 11c black arrows). At day 4, following cells loading, the cells expanded into adjacent vessels (Fig 11e & 11f white arrows). At day 6, cells were seen to be packed into larger vessels, and occupied some of the tubules (Fig 11h & 11i white arrows). Cells cultured on cryostored decellularized kidney scaffolds also showed similar pattern of repopulation when compared with cells cultured on freshly isolated kidney. The increase in their number after additional 3 days of culture thus reiterated that cryopreserved kidneys could be used with same potential as freshly isolated kidneys for recellularization following decellularization.

## Discussion

End-stage renal (ESR) diseases lay an immense stress on human health-care system. It afflicts more than 300,000 individuals in the United States and with an alarmingly large population in third world countries like India. In 2003, more than 2,000 patients received a kidney transplant as a part of their therapy, and the number of patients requiring a kidney transplant has steadily grown. It has been estimated that on average 3,000–3,500 kidney transplants that are occurring every year, barely 5% donated organs come from the brain-dead. The shortage of kidney donors remains an unsolved problem that creates a transplant crisis. Thus, regenerative medicine including cellular therapy combined with tissue engineering for generating functional tissues to tackle the problem of organ shortage is gaining ground. Various research groups have attempted to show that decellularized extracellular matrix scaffold can direct regeneration of the kidney [10–15]. It has inclined the research towards adopting novel approaches for decellularization and recellularization process. The present observations shed a light on this aspect and show that 3 months of cryostorage can well retain the kidneys architecture both pre and post decellularization. The characteristics of the decellularized structure further reiterated the similarity between the de-cellualrized scaffolds as the potential of recellularization using mesenchymal stem cells also remained same whether the scaffolds was generated using freshly isolated kidneys or the cryostored tissues. These observations additionally invoke interest for using MSCs in the renal tissue recellularization program.

As such the cryopreservation technologies have often been overlooked until a construct is very close to being clinically available. The preservation of these constructs during manufacturing processes and transport is critical in bringing these constructs on-the shelf, for the clinical use ([16, 17]. Biologic scaffolds like skin, bone, cartilage, can be cryopreserved for prolonged periods of time prior to use. But not much has been elucidated with respect to cryostorage and subsequent decellularization /recellularization of kidney. Thus, unlike, certain tissues such as blood vessels [1], urinary bladder [2], and trachea [3] for which significant progress has been made to develop and design novel constructs for their repair, replacement or regeneration, the regeneration of kidney demands more rigorous strategies. This is due to the fact that renal tissue is more heterogeneous and composed of ~26 different cell types in a spatial arrangement [18]. It is indeed challenging to achieve a viable decellularized scaffold, more so when it has undergone cryopreservation, for the regeneration process. As it primarily seems to be first ever study to examine decellularization/recellularization of cryostored rat kidney sections, hence, it necessitated an optimization based on comparison between decellularization potential of freshly isolated kidney to that of 3 months of cryostorage. Both the DNA content and preservation of native extracellular components were used for the characterization studies owing to the very nature of the decellularization cocktail used for developing a decellularized structure. It was interesting to note that both the freshly isolated kidney (Group II) as well as 3 months cryostored kidney (Group III), after decellularization with SDS, generated a uniform skeleton with gross appearance of a completely acellular organ. These results are comparable to other studies that have used the similar concentrations and constituents of decellularization cocktail [10,12,13,19] suggesting that de-celluarization of cryostored kidney can be achieved in the similar manner. Almost similar extent of decrease in the DNA content in both types of kidneys, 3 months cryostored (Group III) versus freshly isolated (Group II), thus fulfills the far more important criterion of achieving a residual DNA content to almost negligible amount. For clinical transplantation of decellularized organ this criterion is critically important, wherein any residual DNA fragments have been shown to elicit an immunological response and may be a contributing factor in tissue rejection [8, 20].

The other critical factor being the preservation of the basic cytoskeletal architecture and extracellular matrix protein within these decellularized structures on cryopreservation. Since, these being the key facilitator for the reconstitution of a functional organ we characterized the decellularized scaffolds through histological (H & E, MT, Alcain Blue, PAS stains, Figs 2 to 6) analysis. Both collagen and glycosaminoglycans are necessary components for structural integrity and cellular support [21]. The observations indeed reiterated towards maintenance of extracellular matrix components in microvasculature and within in whole intact renal structures. No significant loss of sulfated glycosaminoglycans, collagen and laminin throughout the decellularized organ was seen upon comparing decellularized structures of cryopreserved kidneys to that of freshly isolated kidneys. Both freshly isolated and cryostored kidney also demonstrated preservation of the native ultrastructure, as could be analyzed through SEM. Many reports lend credibility to the fact that maintaining the basic structural characteristics of different organs upon preservation provides key signals from the aforementioned proteins for all the necessary cues needed for optimal cell reseeding/ recellularization of the decellularized structures [4, 8, 22, 23]. Besides these cues, the functional integrity of the decellularized organ is also a function of the biomechanical strength that these proteins lend to an organ. No doubt bio-mechanical strength of the soft tissues undergoes a drastic variation upon decellularization [11], but it becomes an important parameter specifically when the organ is to be stored for long term before being used in generation of scaffold. The 3 months storage as used in the current study provides a sufficient time period to analyze this effect. A transitional stress bearing ability of the cryostored decellularized structures further lend credibility to restoration of the kidney architecture following cryostorage. It is known that cell shape stability is linked to and depends upon the maintenance of mechanical force balance within the cytoskeleton. Tensional pulling forces that are generated within contractile microfilaments are balanced partly by traction forces exerted on the cell’s external tethers to the ECM substrate, and partly by internal microtubules that resist compression inside the cell. By balancing these forces among microfilaments, microtubules and the ECM, the cell generates a ‘prestress’, or state of isometric tension, in the cytoskeleton that mechanically stabilizes cell shape and regulates cell fate determination [24]. Thus, achieving a level of mechanical strength following cryo-storage and freshly isolated kidneys, even though two fold less in former than the later, provides a proof of concept that even long term cryostorage could be easily exploited for the recellularization studies. This is supported by the fact that in spite of variation in mechanical strength of the decellularized structures, almost all the acellular structures could be well restored to their recelluraized form [4, 25, 26] Thus, as described herein, the overall mechanical strength of decellularized structures, which is far more for freshly decellularized scaffold compared to those from cryostored kidneys, increased when compared to control tissue. This is attributed to the relative less stress due to cellular loss and the reduction of other components (i.e. water) following decellularization. For replenishing the cells, we in the present study, exploited murine C_3_H_10_T½ Cells as these possess the potential of transdifferentiation owing the extracellular matrix (ECM) cues available to these cells [27]. The natural scaffold possibly would provide homing to these cells in the current study. Moreover, several studies have indicated that treatment with MSC can ameliorate severe injury to the renal tissues possibly by replenishing tubular epithelial cells [28], mesangial cells [29–31], podocytes [32, 33], and endothelial cells [34–37]. Likewise many other studies corroborated the potential of MSC’s in renal tissue regeneration [38–48]. These reports suggested that MSCs transplantation can be exploited for replenishing the renal cells. No doubt we do believe that further research is required to evaluate and solve the problems associated with regeneration therapy in order to make safe and effective use of MSC for kidney regeneration. MSC’s being multipotent in nature, these pose relatively less stringent ethics concern, possess immunomodulatory capacities and above all have the tendency to migrate to sites of tissue injury/inflammation and escape immune-recognition [49–53]. However for exploiting the maximum potential of these cells for the recellularization the cell viability is an important aspect owing to the chemical nature of the decellularization cocktail. The excessive washing of the decellularized scaffolds, in the current study, well maintained the viability of the cells when plated on the scaffolds. The histological analysis of the both scaffolds further lend credibility that scaffolds (whether obtained from freshly isolated kidneys or from cryopreserved kidneys for 3 months) were capable of supporting murine C_3_H_10_T½ cells attachment and growth. These results demonstrated the intrinsic capacity of decellularized kidney scaffolds to provide important cues so as to increase cell–cell and cell–ECM interactions to promote cell growth. These observations lend credibility to the fact that cryostored renal tissue can be exploited for renal tissue engineering using mesenchymal stem cells. The use of stem cell types in this study additionally provide basis for exploiting these cell types for renal tissue regeneration. It is certainly evident by the fact that the cues secreted by mesenchymal stem cells are known to direct signaling molecules important for renal tissue re-growth [54]. Taken together, current data establishes foundations for *in vitro* renal tissue engineering with decellularized kidney scaffolds and also brings to fore the utility of cryopreservation for both decellularization and recellularization, and lay the foundations for optimizing the methodologies necessary for eventual *in vivo* transplantation for patients. As a proof of concept, findings from this study serve as an important basis for long term studies that would be utilized for the development of future bioengineered renal constructs from the cryostored cadaveric human tissues.

## Supporting information

S1 Fig The cryopreserved kidneys upon thawing retains the vasculature similar to freshly isolated wild type kidneys and perfusion with 1% SDS completely decellularized the both types of kidneys. The collateral kidneys were isolated either from freshly sacrificed wild type rats or thawed from cryopreserved tissue (3 months). Both the kidneys looked well vascularized. These kidneys were perfused with normal saline prior to perfusion with 1% SDS diluted in distilled water at room temperature for a period of 48 hours. The kidneys were then washed with a solution of deoxyribonuclease I (0.2 mg/ml) and 10 mM MgCl_2_ in PBS at room temperature for a period of 16 h to ensure complete removal of detergents and nuclear material. Finally the structures were rinsed with PBS to remove deoxyribonuclease 1 and MgCl_2_ The photomicrographs represents **a)** control freshly isolated kidney, **b)** thawed kidneys following 3 months cryopreservation, **c)** decellularized freshly isolated kidney and **d)** decellularized kidney following 3 months of cryopreservation. Both the kidney structures whether cryopreserved **(b)** or not **(a)** retain similar level of vascularization. White transparent appearance of both kidneys from **c and d** shows that kidneys have undergone complete loss of parenchyma in comparison to the kidneys whether isolated freshly or following 3 months of cryopreservation oup II and cryostroed kidneys, group III.
(TIF)

S2 Fig Effects of cryopreservation of renal tubules diameter with in medullary region. Photomicrographs represented H&E staining of **a)** control kidneys **b)** decellularized structure from freshly isolated kidney and **c)** decellularized structure from cryostored kidney at 100X. The table below shows that the tubular diameter from medullary region of wild type control kidney **(a)** was highest followed by decellularized structure from cryopreserved kidney **(c)** and the smallest for the decellularized structure from freshly isolated kidney **(b).** This photomicrograph is a representative image out of five separate images those were used for measuring the diameter.
(TIF)

S3 Fig Effects of cryopreservation of renal glomerulur diameter. Photomicrogrpahs **a-c)** showed the Masson’s trichrome stained images, this dye stains collagen blue, cellular component red and nuclei black. The image showed well retained collagen component of the ECM from **a)** represented control kidney **b)** decellularized structure from freshly isolated kidney and **c)** decellularized structure from 3 months cryostored kidney group. These images were used to quantify the diameter of the glomeruli from wild type control kidneys and dec-llularized kidneys from freshly isolated tissue and 3 months cryopreserved kidneys. The control kidney showed ~ 115.3 μm diameter followed by 79.4 μm for decellularized structure from cryopreserved kidney and about 54.54 μm for decellularized structure from freshly isolated kidney.
(TIF)
