# Supplementary figures and images for: Decellularized scaffold of cryopreserved rat kidney retains its recellularization potential
Source: PLoS One. 2017 Mar 7;12(3):e0173040. doi: 10.1371/journal.pone.0173040 (PMC5340383; doi:10.1371/journal.pone.0173040)

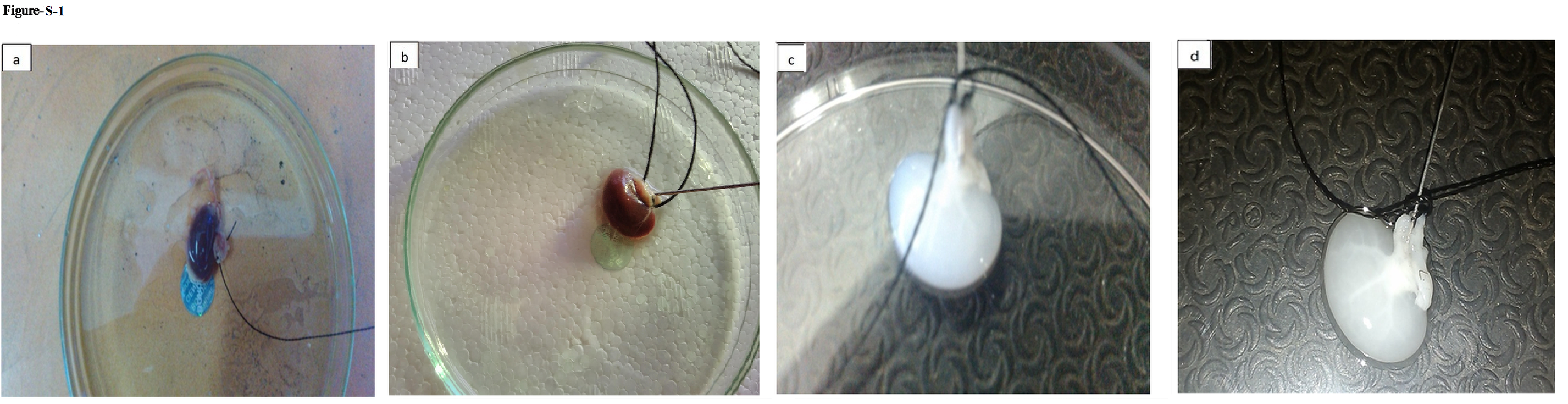

Supplement: S1 Fig — The collateral kidneys were isolated either from freshly sacrificed wild type rats or thawed from cryopreserved tissue (3 months). Both the kidneys looked well vascularized. These kidneys were perfused with normal saline prior to perfusion with 1% SDS diluted in distilled water at room temperature for a period of 48 hours. The kidneys were then washed with a solution of deoxyribonuclease I (0.2 mg/ml) and 10 mM MgCl2 in PBS at room temperature for a period of 16 h to ensure complete removal of detergents and nuclear material. Finally the structures were rinsed with PBS to remove deoxyribonuclease 1 and MgCl2 The photomicrographs represents a) control freshly isolated kidney, b) thawed kidneys following 3 months cryopreservation, c) decellularized freshly isolated kidney and d) decellularized kidney following 3 months of cryopreservation. Both the kidney structures whether cryopreserved (b) or not (a) retain similar level of vascularization. White transparent appearance of both kidneys from c and d shows that kidneys have undergone complete loss of parenchyma in comparison to the kidneys whether isolated freshly or following 3 months of cryopreservation oup II and cryostroed kidneys, group III. (TIF) [file pone.0173040.s001.tif]

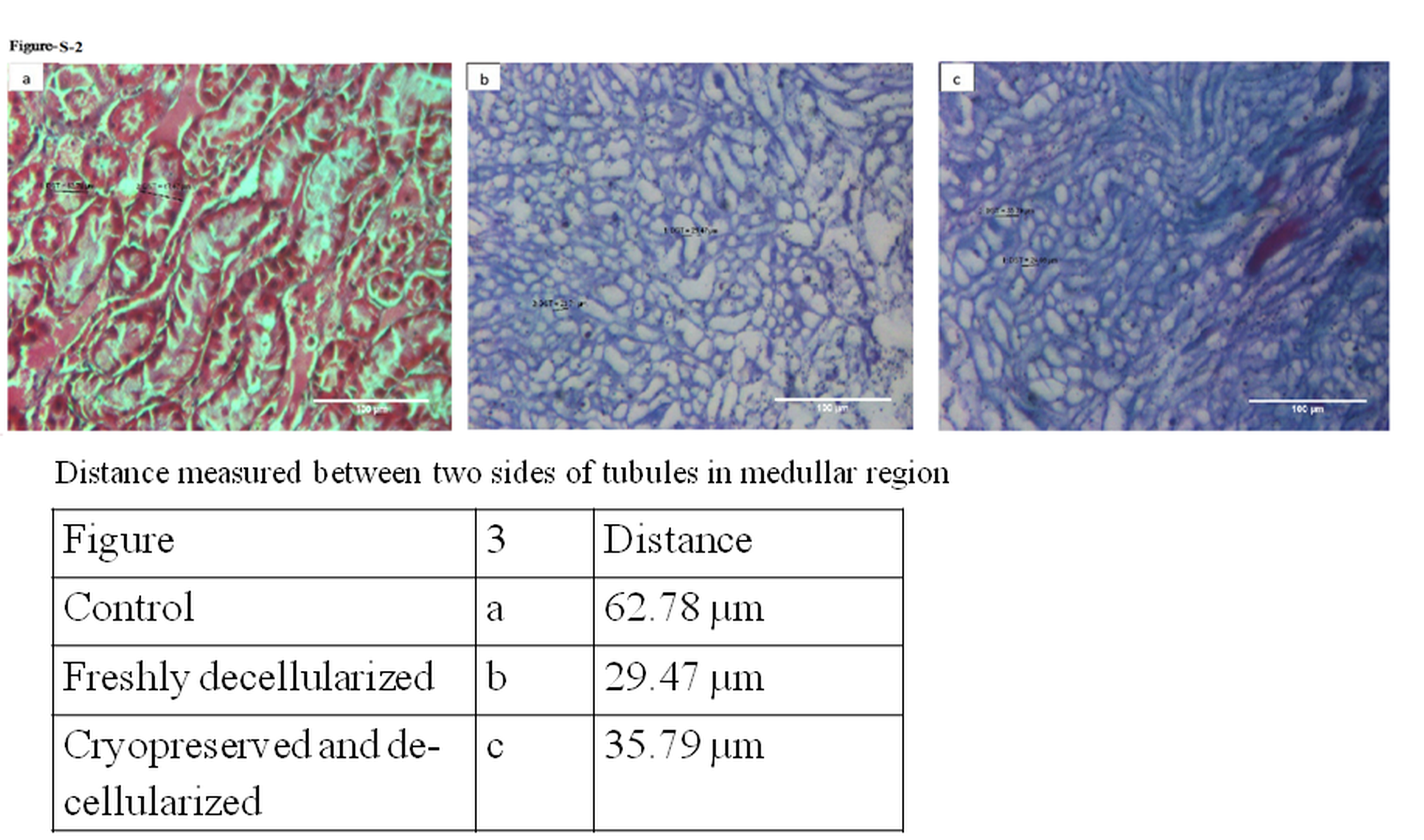

Supplement: S2 Fig — Photomicrographs represented H&E staining of a) control kidneys b) decellularized structure from freshly isolated kidney and c) decellularized structure from cryostored kidney at 100X. The table below shows that the tubular diameter from medullary region of wild type control kidney (a) was highest followed by decellularized structure from cryopreserved kidney (c) and the smallest for the decellularized structure from freshly isolated kidney (b). This photomicrograph is a representative image out of five separate images those were used for measuring the diameter. (TIF) [file pone.0173040.s002.tif]

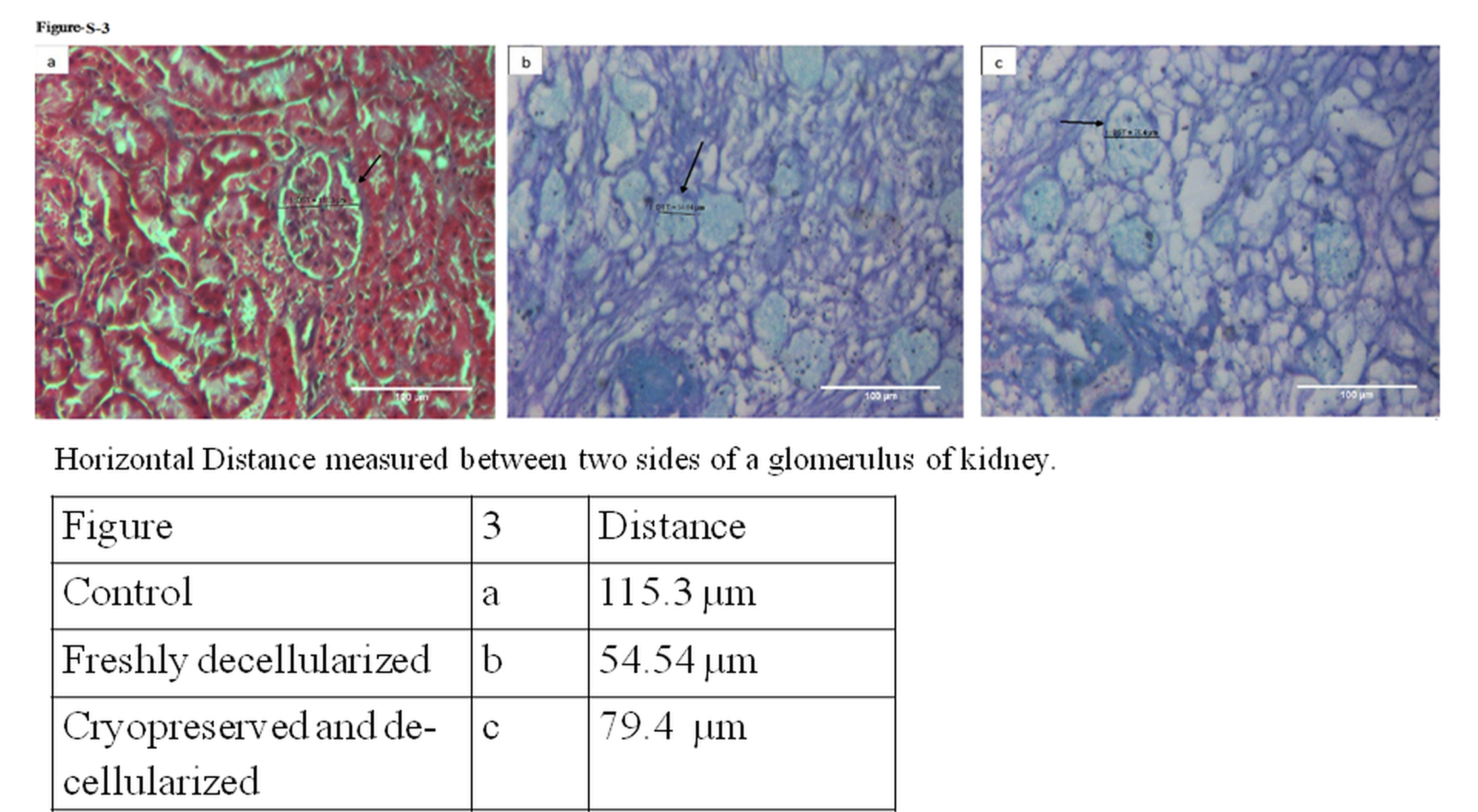

Supplement: S3 Fig — Photomicrogrpahs a-c) showed the Masson’s trichrome stained images, this dye stains collagen blue, cellular component red and nuclei black. The image showed well retained collagen component of the ECM from a) represented control kidney b) decellularized structure from freshly isolated kidney and c) decellularized structure from 3 months cryostored kidney group. These images were used to quantify the diameter of the glomeruli from wild type control kidneys and dec-llularized kidneys from freshly isolated tissue and 3 months cryopreserved kidneys. The control kidney showed ~ 115.3 μm diameter followed by 79.4 μm for decellularized structure from cryopreserved kidney and about 54.54 μm for decellularized structure from freshly isolated kidney. (TIF) [file pone.0173040.s003.tif]
